# Shared War reality effects on the professional quality of life of mental health professionals

**DOI:** 10.1186/s13584-016-0075-6

**Published:** 2016-08-15

**Authors:** Itay Pruginin, Dorit Segal-Engelchin, Richard Isralowitz, Alexander Reznik

**Affiliations:** 1 Spitzer Department of Social Work and the Regional Alcohol and Drug Abuse Research Center (RADAR) , Ben-Gurion University of the Negev, POB 653, Beer-Sheva, 84105 Israel; 2 Spitzer Department of Social Work and Center for Women’s Health Studies and Promotion, Ben- Gurion University of the Negev, POB 653, Beer-Sheva, 84105 Israel

**Keywords:** Mental health professionals, Shared war reality, Trauma exposure levels, Professional quality of life

## Abstract

**Background:**

To date, studies on the outcomes of a shared war reality among mental health professionals (MHPs) in southern Israel have focused only on those residing and working in Otef Gaza. The aim of this study is to determine the impact of different exposure levels to shared trauma on the professional quality of life of MHPs in southern Israel. This study compares the level of secondary traumatic stress, burnout, and compassion satisfaction of social workers from Otef Gaza to social workers living and working in the Beer-Sheva area who experience occasional missile attacks.

**Methods:**

The Professional Quality of Life Scale was used to examine the level of secondary traumatic stress, burnout, and compassion satisfaction of 125 social workers living and working in the Negev: 72 from Beer-Sheva and 53 from the regional councils of Otef Gaza.

**Results:**

No statistically significant differences were found in the three professional quality of life variables between the Otef-Gaza and Beer-Sheva groups.

**Conclusions:**

The lack of secondary traumatic stress and burnout differences between the study groups, despite the chronic exposure to terror attacks among the Otef Gaza social workers, may be explained by the strong sense of belonging and support evidenced by many Otef Gaza residents as well as by the comprehensive trauma training MHPs receive for work in the region. The results of this study are important for health policy geared to trauma prevention efforts, moderating the effects of work under shared war reality, and promoting the professional quality of life of MHPs in conflict areas.

## Background

Since 2001, people living in the southern region of Israel have suffered from missile attacks from Gaza. The Israeli area bordering Gaza known as “Otef Gaza,” consisting of five regional councils - Sderot, Hof Ashkelon, Sdot Negev, Sha’ar Hanegev and Eshkol, has been most affected. Among the Otef Gaza regional councils only Sderot with 24,000 inhabitants may be considered an urban community. The others are comprised of small rural collective settlements.

In September 2005, Israel disengaged from Gaza resulting in the removal of its people - civilian and soldiers, private residences, community facilities and military bases. Since that time more than 11,600 missiles have been fired from Gaza, most hitting Otef Gaza. During the 2014 conflict alone, known as Operation “Protective Edge”, more than 4,500 missiles were fired from Gaza. While most Otef Gaza residents “stood their ground,” an estimated 10,000 civilians left the area [1].

In Otef Gaza, as well as elsewhere in the country, disaster mental health service is a shared responsibility addressed by local communities, national government components, and non-government organizations modified over time through “true lessons observed and learned” [2]. Mental health professionals (MHPs) are among the first responders to address the needs of traumatized people following exposure to terrorist attacks and war-related stressors. In Otef Gaza, MHPs encounter a double exposure to war-related trauma as community members and professionals providing service to terror victims [3]. This phenomenon of coping with the same trauma as their clients is referred to as ‘shared trauma’. Consequently, MHPs may be subject to increased vulnerability [4] that affects their personal well-being and professional confidence. In a study of Israeli emergency room social workers in northern Israel, those from communities subject to terror attacks were found to have a lost sense of personal security and professional detachment needed to function under difficult conditions [5]. Research of MHPs living and working in Otef Gaza during the Gaza War in 2008-2009 shows that shared trauma conditions are related to increased emotional pressure, family conflict [6], parenting problems [7], and an increased risk for PTSD and vicarious symptoms. [8] Also, increased levels of PTSD symptoms have been found among physicians and nurses exposed to shared trauma during the Gaza War in 2008–2009 [9].

Much research has focused on the negative outcomes of shared trauma; however, positive effects exist as well [10]. These include compassion satisfaction that results from helping others and positive collegial relations [11–13]. This outcome may serve as a protective factor by strengthening the sense of worthiness [14] and decreasing of burnout vulnerability [12] of MHPs. Post-traumatic growth [15] is another positive outcome of shared trauma resulting from successfully overcoming a difficult or traumatic event. Helping others overcome traumatic events results in satisfaction and increased levels of self- knowledge and self-awareness to the caregiver [15]. Also, studies of professionals who share war-related reality with their clients show the negative effects of shared trauma can be accompanied by positive effects such as personal and professional growth [3, 16–18].

To date, studies on the outcomes of shared trauma situations among MHPs in southern Israel have focused only on those living and working in Otef Gaza. The aim of this study is to determine the impact of different exposure levels to shared trauma on the professional quality of life of social workers in southern Israel. This study compares levels of secondary traumatic stress, burnout, and compassion satisfaction among social workers from Otef Gaza, who have experienced missile attacks for more than a decade with only 15 s to reach shelter following a warning siren, and those living and working in the Beer Sheva area who tend to have less shared trauma experience resulting from sporadic missile attacks from Gaza and large military operations (e.g., Operation “Cast Lead,” 2008; Operation “Pillar of Defense,” 2012; Operation “Protective Edge”, 2014) with up to 60 s to reach a shelter [1].

Given the key role MHPs have addressing emergencies resulting from armed conflicts and terror attacks, it is important to identify conditions and factors that promote their ability to function in shared trauma situations. Such findings provide valuable insight of health policy needed for trauma prevention and professional quality of life maintenance of MHPs operating in conflict areas.

## Methods

### Sample and procedure

The study cohort consists of 125 MHPs living and working in the Negev: 72 from Beer Sheva, and 53 from the regional councils of Otef Gaza – the majority (93 %) living and working in Otef Gaza. All MHPs were social workers.

The study was conducted one month after the 2012 Operation Pillar of Defense that lasted eight days. The study and information collection process was approved by the Israel Ministry of Welfare and Social Services and the Ben Gurion University – Department of Social Work Ethics Committee. The managers of the welfare departments in Beer-Sheva and the regional councils of Otef Gaza were informed of the study goals and procedure by the researchers, and were then asked to inform their staff about the study. Self-report questionnaires were administered during the welfare departments’ monthly staff meetings. Among the social workers involved with this study, ninety-seven per cent from Beer-Sheva and ninety-five percent from the Otef Gaza regional councils agreed to participate and completed informed consent forms.

### Measures

#### Sociodemographic information

The sociodemographic questions asked about gender, age, marital status, level of education, degree of religious observance (according to three categories: (1) religious; (2) traditional; and (3) secular), years of professional experience and number of children.

#### The professional quality of life scale

Participants’ levels of secondary traumatic stress, burnout, and compassion satisfaction were assessed using the Professional Quality of Life Scale (ProQOL5, [19]). This 30-item scale was developed to measure the negative and positive effects of helping others who experience suffering and trauma. The ProQOL has three 10-item sub-scales: compassion satisfaction (e.g., “I get satisfaction from being able to [help] people”); burnout (e.g., “I feel trapped by my job as a helper]”); and, secondary traumatic stress (e.g., “I feel as though I am experiencing the trauma of someone I have [helped]”). Participants were asked to rate how frequently they experienced each of the 30 items within the context of their work situation during the last 30 days on a 5-point Likert scale, ranging from 1 (never) to 5 (very often). Scores for each subscale range from 10–50 with scores categorized as follows: low - 22 or less; average - between 23–41; high – 42 or more [19]. The three sub-scales have been found to have good reliability (Compassion Satisfaction α = .88; Burnout α = .75; Secondary Traumatic Stress α = .81) [19]. In the current sample, Cronbach’s alpha reliability coefficients were found to be .84, .64.and .74 for Compassion Satisfaction, Burnout, and Secondary Traumatic Stress, respectively. Previous findings indicate that the ProQOL scale is capable of distinguishing between different groups of health care providers [20, 21].

### Statistical analyses

Chi-square tests were carried out to examine differences between the two groups on the categorical demographic variables. Descriptive statistics were used to examine means and standard deviations of the ordinal demographic variables and professional quality of life measures. T tests were conducted to examine differences in ordinal demographic variables and in professional quality of life variables between the two groups.

## Results

Table 1 presents the distribution (*n*, %) of the two research groups by gender, marital status, education and religious observance as well as the means and the standard deviations of age, number of children and years of professional experience.

**Table 1:** Demographic Description of the Two Groups (*N* = 125)

	Beer Sheva (*N* = 72)	Otef Gaza (*N* = 53)	df	*χ* ^2^/*t* test
Gender				
Male, % (*n*)	5.6 (4)	19.2 (10)	1	5.63*
Female, % (*n*)	94.4 (68)	80.8 (42)		
Age, Mean (SD)	41.6 (12.3)	42.2 (11.1)	121	0.27
Children, Mean (SD)	2.6 (1.6)	2.8 (1.2)	104	0.94
Years of professional Experience, Mean (SD)	13.0 (10.6)	12.8 (9.2)	120	0.09
Marital status				
Married/Partnered, % (*n*)	75.0 (54)	86.8 (46)	1	2.65
Other, % (*n*)	25.0 (18)	13.2 (7)		
Education				
BA, % (*n*)	65.3 (47)	56.6 (30)	1	0.97
MA, % (*n*)	34.7 (25)	43.4 (23)		
Religious Observance				
Secular % (*n*)	48.6 (35)	49.1 (26)	2	0.46
Traditional % (*n*)	22.2 (16)	26.4 (14)		
Religious % (*n*)	29.2 (21)	24.5 (13)		

*p < .05

As shown in Table 1, chi-square tests reveal no statistically significant difference between the Otef-Gaza and Beer Sheva groups in marital status, education level and degree of religious observance. For both groups, most participants were married, secular and many of them had a graduate degree in social work. A statistically significant difference was found in the gender status of the participants- the Otef-Gaza group had more male social workers than the Beer-Sheva group. T-tests revealed no statistically significant difference between the groups in terms of participants’ age, number of children, and years of professional experience.

Table 2 shows the mean value, standard deviation and *t* test results comparing the three professional quality of life variables. As shown in Table 2, no significant differences were found between the two study groups. According to the ProQOL cut-off scores [19], the social workers in both groups had average levels of compassion satisfaction, burnout and secondary traumatic stress.

**Table 2:** Means and Standard Deviations for Professional Quality of Life Measures

Professional Quality of Life Measures	Beer Sheva (*N* = 72)		Otef Gaza (*N* = 53)		*t*-test
	M	SD	M	SD	
Compassion Satisfaction	38.6	(4.1)	38.7	(5.3)	0.16 (ns)
Burnout	22.8	(3.7)	23.1	(4.8)	0.36 (ns)
Secondary Traumatic Stress	22.4	(4.9)	22.8	(5.3)	0.40 (ns)

## Discussion

The importance of identifying the negative and positive effects of shared trauma among MHPs operating in conflict areas was the impetus for the current study. The study examined whether shared trauma outcomes are affected by the frequency and intensity of missile attack exposure. Specifically, the comparison of social workers from different areas of southern Israel provided the opportunity to examine the effects of missile attack exposure on three components of professional quality of life: secondary traumatic stress, burnout, and compassion satisfaction.

The findings of this study indicate no significant differences in the three components of professional quality of life between social workers from Otef Gaza and the Beer-Sheva area. The lack of such differences between the two study groups is consistent with other findings that show the number of therapist trauma-focused clients is not a predictor of secondary traumatic stress [10]. Also, the present study results are consistent with other findings that show subjective exposure to terror attacks, reflected by perceived threat rather than actual exposure, is a significant predictor of PTSD and vicarious trauma symptoms among MHPs [8]. A possible explanation for our findings, requiring further investigation, is that being exposed to repetitive missile attacks for over a decade Otef Gaza social workers may feel less threatened by terror events and more able to assist terror victims in their community than their Beer-Sheva counterparts who experience occasional missile attacks. In other words, the higher level of exposure to terror attacks and terror victims paradoxically may have a moderating effect on the impact of the shared war reality among Otef-Gaza social workers by increasing their ability to cope with war-related stressors on personal and professional levels. Thus, although they have experienced more frequent and intense missile attacks, they did not report higher levels of distress than the Beer-Sheva MHPs.

Another possible explanation for the similarities among the study groups may be related to the large amount of resources invested in Otef Gaza during the past 15 years in order to cope with the war-related reality. A key element aimed to increase resilience among Otef Gaza inhabitants was the establishment of ‘Resilience Centers’ aimed at increasing civic resilience on individual, communal and municipal levels. These centers train volunteer and professional caregivers who work with terror victims in the region [22]. The training encompasses multiple trauma treatment interventions including Eye Movement Desensitization Reprocessing, Somatic Experience, Sensory Ethical Extrovert, Cognitive Behavioral Therapy and other methods [23]. Professional support has indeed been found to moderate the relationship between exposure and distress among mental health professionals working and residing in areas exposed to frequent terrorist attacks [8]. Further research is needed on the role that training and professional support play in shaping the experience of social workers working in a shared war reality.

A third possible explanation for the lack of difference between the groups, despite the higher level of exposure to terror attacks among Otef Gaza social workers, may be related to the nature of their communities. Beer Sheva is the seventh largest city in Israel with 201,000 inhabitants [24], where most communities in Otef Gaza (with the exception of Sderot) are relatively small rural enclaves. Research indicates that small rural communities may have enhanced resilience to trauma [25, 26]. This is due their relatively high levels of social cohesion, strong and wide social support networks, and strong social institutions. Residents of Otef Gaza rural communities have been found to have a strong sense of belonging, community solidarity, and confidence in the state authorities (i.e., police, Israel Defense Forces, etc.) suggesting these characteristics may be protective factors for the local population [27]. These same factors may have also served as a buffer from the repercussions of work under shared war reality.

Other studies show maturity [10] and years of clinical experience [14] are positive predictors of compassion satisfaction among trauma treatment therapists. Thus, the lack of difference between the two research groups in compassion satisfaction may be related to age and professional experience similarities.

The current study has several limitations. First, the sample consists solely of social workers employed by the municipalities and regional councils in southern Israel. Further inquiry should include social workers employed by different agencies (e.g. NGO’s and private clinics) and additional healthcare providers such as physicians and nurses. Second, the cross-sectional design of the study does not contribute to determination of the long-term impact of the shared trauma on the professional quality of life of social workers. Future studies may benefit from investigating the long-term outcomes of different exposure levels among MHPs in conflict areas. Another methodological issue that should be taken into account when interpreting the findings is that the study was conducted a month after Operation Pillar of Defense. Different findings may have been obtained had the study been conducted during the course of the operation when residents of Otef Gaza encountered heavy bombardment and deeper disruption of daily living than those residing in Beer-Sheva.

Further research is needed to validate these findings both in Israel, in different locations, including those that are subject to lower levels of missile attacks (e.g., Tel Aviv, Jerusalem and northern Israel), and other countries so that such information may be useful for mental health professional policy purposes and service provision as well as the training of practitioners addressing the treatment needs of people affected by disaster and terror attacks. Comparison of the professional quality of life of Israeli MHPs and of their international counterparts, operating in similar settings, would enable us to explore the role of culture in resilience.

Also, future studies would benefit from examining whether the professional quality of life of social workers living and working in Otef Gaza and in Beer-Sheva is related to their years of employment in these areas, an issue that was not addressed in the current study. Lastly, qualitative studies are recommended in order to provide a more detailed understanding of the unique experience of working in a shared war-related reality, over time and across locations.

## Conclusions

In conclusion, our results indicate that the outcomes of work under shared war reality among social workers are not related to the frequency and intensity of exposure to the missile attacks. The results raise the possibility, subject to additional research, that a strong sense of belonging, terrorism preparedness and extensive training in trauma treatment interventions may serve as protective factors for the social workers operating in Otef Gaza. The major implication of this study is the importance of preparing social workers to cope with the challenges and complexities of work under shared war reality and providing assistance to terror victims. This may be done by incorporating courses on war-related trauma treatment interventions into the curriculum of undergraduate social work programs as well as by developing advanced courses for qualified social workers focusing on evidence-informed interventions for terror victims.

Given the paucity of comparative studies on MHPs operating under shared war reality across locations in the country (e.g., Otef Gaza, Beer Sheva, Tel Aviv and elsewhere), this paper makes an initial contribution to understanding shared war reality effects on the professional quality of life of MHPs.

## References

[1] Israel Ministry of Foreign Affairs. The 2014 Gaza conflict: Factual and legal aspects. http://mfa.gov.il/protectiveedge/pages/default.aspx (2015). Accessed 10 Oct 2015.

[2] Schreiber MD (2015). Toward the way forward: Building an emergency mental health system for Israel. Isr J Health Policy Res.

[3] Baum N (2014). Professionals’ double exposure in the shared traumatic reality of wartime: Contributions to professional growth and stress. Brit J Soc Work.

[4] Saakvitne K (2002). Shared trauma: the therapists’ increased vulnerability. Psychoanal Dialogues.

[5] Somer E, Buchbinder E, Peled-Avram M, Ben-Yizhack Y (2004). The stress and coping of Israeli emergency room social workers following terrorist attacks. Qual Health Res.

[6] Baum N (2012). Emergency routine: The experience of professionals in a shared traumatic reality of war. Brit J Soc Work.

[7] Dekel R, Nuttman-Shwartz O, Pat-Horenczyk R, Brom D, Chemtob C, Vogel J (2014). Being a parent and a helping professional in the continuous shared traumatic reality in southern Israel. Helping children cope with trauma: Individual, family and community perspectives.

[8] Finklestein M, Stein E, Greene T, Bronstein I, Solomon Z (2015). Posttraumatic stress disorder and vicarious trauma in mental health professionals. Health Soc Work.

[9] Ben-Ezra M, Palgi Y, Wolf JJ, Shrira A (2011). Psychiatric symptoms and psychosocial functioning among hospital personnel during the Gaza War: a repeated cross-sectional study. Psychiatry Res.

[10] Sodeke-Gregson EA, Holttum S, Billings J. Compassion satisfaction, burnout, and secondary traumatic stress in UK therapists who work with adult trauma clients. Eur J Psychotraumatol. 2013; 4. doi: 10.3402/ejpt.v4i0.21869 10.3402/ejpt.v4i0.21869 PMC3877781 24386550

[11] Boscherino AJ, Figley RC, Adams ER (2004). Compassion fatigue following the September 11 terrorist attack: a study of secondary trauma among New York city social workers. Int J Emerg Ment Health.

[12] Conrad D, Kellar-Guenther Y (2006). Compassion fatigue, burnout, and compassion satisfaction among Colorado child protection workers. Child Abuse Negl.

[13] Figley CR (2002). Treating compassion fatigue.

[14] Craig CD, Sprang G (2010). Compassion satisfaction, compassion fatigue, and burnout in a national sample of trauma treatment therapists. Anxiety Stress Coping.

[15] Calhoun LG, Tedeschi RG (1999). Facilitating posttraumatic growth: A clinician guide.

[16] Dekel R, Baum N (2010). Intervention in a shared traumatic reality: A new challenge for social workers. Brit J Soc Work.

[17] Lev-Wiesel R, Goldblat H, Eisikovits Z, Admi H (2008). Growth in the shadow of war: the case of social workers and nurses working in a shared war reality. Brit J Soc Work.

[18] Shamai M, Ron P (2009). Helping direct and indirect victims of national terror: Experiences of Israeli social workers. Qual Health Res.

[19] Stamm BH (2010). The Concise ProQOL Manual.

[20] Avieli H, Ben-David S, Levy I (2016). Predicting professional quality of life among professional and volunteer caregivers. Psychol Trauma.

[21] Bellolio MF, Cabrera D, Sadosty AT, Hess EP, Campbell RL, Lohse CM, Sunga KL (2014). Compassion fatigue is similar in emergency medicine residents compared to other medical and surgical specialties. West J Emerg Med.

[22] Bodas M, Ben-Gershon B, Rubinstein Z, Bergman-Levy T, Peleg K (2015). The evolution of the emergency mental health system in Israel - from the 1980’s until today. Isr J Health Policy Res.

[23] Israel Trauma Coalition. 2012 Annual report: Resilience centers Sderot and Gaza Region (Hebrew). http://www.israeltraumacoalition.org/_Uploads/dbsAttachedFiles/annual_report2012_short_heb.pdf. Accessed 24 Oct 2015.

[24] Israel Central Bureau of Statistics. Press release: Localities and population in Israel 2014 (Hebrew). Jerusalem: Author. 2015. http://www.cbs.gov.il/reader/newhodaot/hodaa_template.html?hodaa=201501279. Accessed 24 Oct 2015.

[25] Kaplan Z, Matar MA, Kamin R, Sadan T, Cohen H (2005). Stress-related responses after 3 years of exposure to terror in Israel: are ideological-religious factors associated with resilience?. J Clin Psychiatry.

[26] Norris FH, Stevens SP, Pfefferbaum B, Wyche KF, Pfefferbaum RL (2008). Community resilience as a metaphor, theory, set of capacities, and strategy for disaster readiness. Am J Community Psychol.

[27] Gelkopf M, Berger R, Bleich A, Silver RC (2012). Protective factors and predictors of vulnerability to chronic stress: A comparative study of 4 communities after 7 years of continuous rocket fire. Soc Sci Med.

